# Privacy-preserving architecture for providing feedback to clinicians on their clinical performance

**DOI:** 10.1186/s12911-020-01147-5

**Published:** 2020-06-22

**Authors:** Kassaye Yitbarek Yigzaw, Andrius Budrionis, Luis Marco-Ruiz, Torje Dahle Henriksen, Peder A. Halvorsen, Johan Gustav Bellika

**Affiliations:** 1 Norwegian Centre for E-health Research, University Hospital of North Norway, 9019 Tromsø, Norway; 2 Department of Community Medicine, Faculty of Health Sciences, UiT The Arctic University of Norway, 9037 Tromsø, Norway; 3 Department of Clinical Medicine, Faculty of Health Sciences, UiT The Arctic University of Norway, 9037 Tromsø, Norway

**Keywords:** Learning healthcare system, Feedback, Peer comparison, privacy, Security, Antibiotic prescriptions, Quality improvement

## Abstract

**Background:**

Learning from routine healthcare data is important for the improvement of the quality of care. Providing feedback on clinicians’ performance in comparison to their peers has been shown to be more efficient for quality improvements. However, the current methods for providing feedback do not fully address the privacy concerns of stakeholders.

**Methods:**

The paper proposes a distributed architecture for providing feedback to clinicians on their clinical performances while protecting their privacy. The indicators for the clinical performance of a clinician are computed within a healthcare institution based on pseudonymized data extracted from the electronic health record (EHR) system. Group-level indicators of clinicians across healthcare institutions are computed using privacy-preserving distributed data-mining techniques. A clinician receives feedback reports that compare his or her personal indicators with the aggregated indicators of the individual’s peers. Indicators aggregated across different geographical levels are the basis for monitoring changes in the quality of care.

The architecture feasibility was practically evaluated in three general practitioner (GP) offices in Norway that consist of about 20,245 patients. The architecture was applied for providing feedback reports to 21 GPs on their antibiotic prescriptions for selected respiratory tract infections (RTIs). Each GP received one feedback report that covered antibiotic prescriptions between 2015 and 2018, stratified yearly. We assessed the privacy protection and computation time of the architecture.

**Results:**

Our evaluation indicates that the proposed architecture is feasible for practical use and protects the privacy of the patients, clinicians, and healthcare institutions. The architecture also maintains the physical access control of healthcare institutions over the patient data. We sent a single feedback report to each of the 21 GPs. A total of 14,396 cases were diagnosed with the selected RTIs during the study period across the institutions. Of these cases, 2924 (20.3%) were treated with antibiotics, where 40.8% (1194) of the antibiotic prescriptions were narrow-spectrum antibiotics.

**Conclusions:**

It is feasible to provide feedback to clinicians on their clinical performance in comparison to peers across healthcare institutions while protecting privacy. The architecture also enables monitoring changes in the quality of care following interventions.

## Background

### Introduction

Rapid learning from data generated in routine clinical care is a major driver of quality improvements. Continuous analysis of routine health data and the provision of feedback to clinicians using reports, decision support systems, or other feedback mechanisms represent the key ideas behind the learning healthcare system paradigm [1–3].

The feedback that compares the performance of clinicians with their peers is even more important for the behavioral change of clinicians [4–8]. Meeker et al. [5] found peer comparison to be the most efficient feedback method to minimize inappropriate antibiotic prescriptions. A large study on the perception of feedback on clinical performance by physicians revealed the importance of peer comparisons with similar patient populations and practice characteristics [6]. Continuous feedback is necessary for sustaining the achieved gains after improvement takes place [4, 9, 10]. However, legitimate privacy concerns are not fully addressed in the current studies.

### Health data reuse

Health data reuse has enormous potential to benefit society by providing healthcare quality improvement [1–3, 11]. However, it raises legitimate privacy concerns from different stakeholders. The reuse of health data is guided by a variety of controls from both legal and ethical perspectives, such as privacy rights, data protection regulations, and duties of confidentiality [12]. However, legal measures pose challenges for the effective reuse of clinical data [13, 14].

Data protection regulations often exempt patient consent for reuse of health data for public health and quality improvement purposes [15, 16]. However, clinicians are often reluctant to pursue it due to the privacy concerns of their patients, themselves, and their institutions [17]. Therefore, data reuse infrastructure must provide solutions for performing data-driven analytics while ensuring the security and privacy of the people and organizations that the data represent [18].

### Privacy-preserving distributed data mining

Privacy-preserving distributed data mining (PPDDM) is an emerging approach for processing data distributed across multiple data sources while protecting privacy [19–22]. Moreover, PPDDM considers the problem of running statistical algorithms on confidential data divided among two or more different parties where an algorithm runs on the combined data of the parties’ databases without allowing any party to view the private data of another party. The statistics generated from the combined data for a group of healthcare institutions are revealed, which are not sensitive information. The PPDDM techniques are developed based on a set of techniques called secure multi-party computation [23].

The existing PPDDM protocols for computing statistical problems provide different privacy guarantees, efficiency, and scalability [19–22]. Secure protocols often provide privacy guarantee against semi-honest (honest-but-curious) adversaries because the adversarial model allows the designing of secure protocols that are efficient and scalable compared to protocols that are secure against other adversarial models that provide a stronger privacy guarantee [23].

The semi-honest adversarial model assumes that even if corrupted parties follow a computation protocol specification, the adversary may try to use the internal state of the corrupted parties, including the messages exchanged during the protocol execution, to learn private information of other uncompromised parties. The adversarial model is suitable for settings in which parties are trusted to follow a computation protocol specification but must run a secure protocol because of legal restrictions for data sharing. This model is also useful to prevent accidental leakage. The security guarantee of this model also ensures that an adversary that gained access to a party’s database after the execution of a secure protocol cannot learn private information about legitimate parties [23]. Therefore, the adversarial model provides a sufficient privacy guarantee in our context while enabling efficient and scalable computation.

Promising progress has been made in protocol design and the implementation of cryptographic primitives fueled by the constant increase in computing power. Protocols specialized for specific statistical problems have been developed to improve the protocols efficiency and scalability, in contrast to the earlier idea of developing generic solutions. Practical uses of PPDDM exist in healthcare settings [24–26]; however, these are still limited primarily due to the lack of efficiency and scalability required for processing health data in practice.

### Use case

The increasing emergence of antibiotic resistance accounts for 700,000 deaths every year worldwide, which is primarily caused by high and inappropriate consumption of antibiotics [27]. Unless actions are taken, the number is estimated to increase [28].

International (e.g., the World Health Organization [29]) and national (e.g., the Norwegian government [30]) initiatives aim to reduce antibiotic use and resistance. Numerous indicators have been proposed for measuring antibiotic prescriptions [31–34]. We refer to Additional file 1 for a detailed description of indicators.

In Norway, a GP is responsible for a set of patients and is expected to diagnose and treat a major proportion of the patients and refer only those who are in need of more specialized health services to hospitals for further assessment and treatment [35]. Thus, Norwegian GPs are responsible for approximately 80% of antibiotic prescriptions for human use [36]. Therefore, behavioral changes by GPs have a significant effect on reducing inappropriate antibiotic prescriptions.

### Objective

The objective of the paper is to propose a distributed architecture for providing feedback to clinicians on their clinical performance in comparison to peers across multiple healthcare institutions while protecting the privacy of the involved patients, clinicians, and healthcare institutions. The architecture also aims to support monitoring antibiotic prescriptions at the group level. We aim to evaluate the feasibility of the architecture in clinical settings using antibiotic prescriptions as a use case.

The proposed architecture uses a PPDDM tool, called Emnet [37, 38], for computing aggregated performance indicators of peers across multiple healthcare institutions while protecting privacy. The architecture also uses a message-oriented middleware, called the Snow system, for secure communication and coordination among involved entities [39].

## Methods

### Privacy requirements and assumptions

All data stored at a healthcare institution are potentially sensitive. Therefore, information about patients, clinicians, and healthcare institutions cannot be disclosed outside the organization that originally recorded the data (*requirement 1*). Aggregated information about a clinician, such as performance indicators, is also private information and should only be accessed by the clinician (*requirement 2*). However, statistics generated from the combined data on a group of healthcare institutions are not considered sensitive information and, thus, can be disclosed. This includes aggregated indicators for a group of clinicians from multiple healthcare institutions.

We assume outside adversaries may compromise a subset of healthcare institutions by breaking into the network or compromising employees. Parties under the control of an adversary are called corrupted parties. We relied on a standard security assumption called the semi-honest (honest-but-curious) adversarial model (*requirement 3*).

We also assumed the existence of a third party, denoted as the *coordinator*, that satisfies the honest-but-curious adversarial model. The coordinator aids computations without learning private information (*requirement 4*). We also assume all the entities have a peer-to-peer secure communication channel.

### Architectural design

**Fig. 1:**
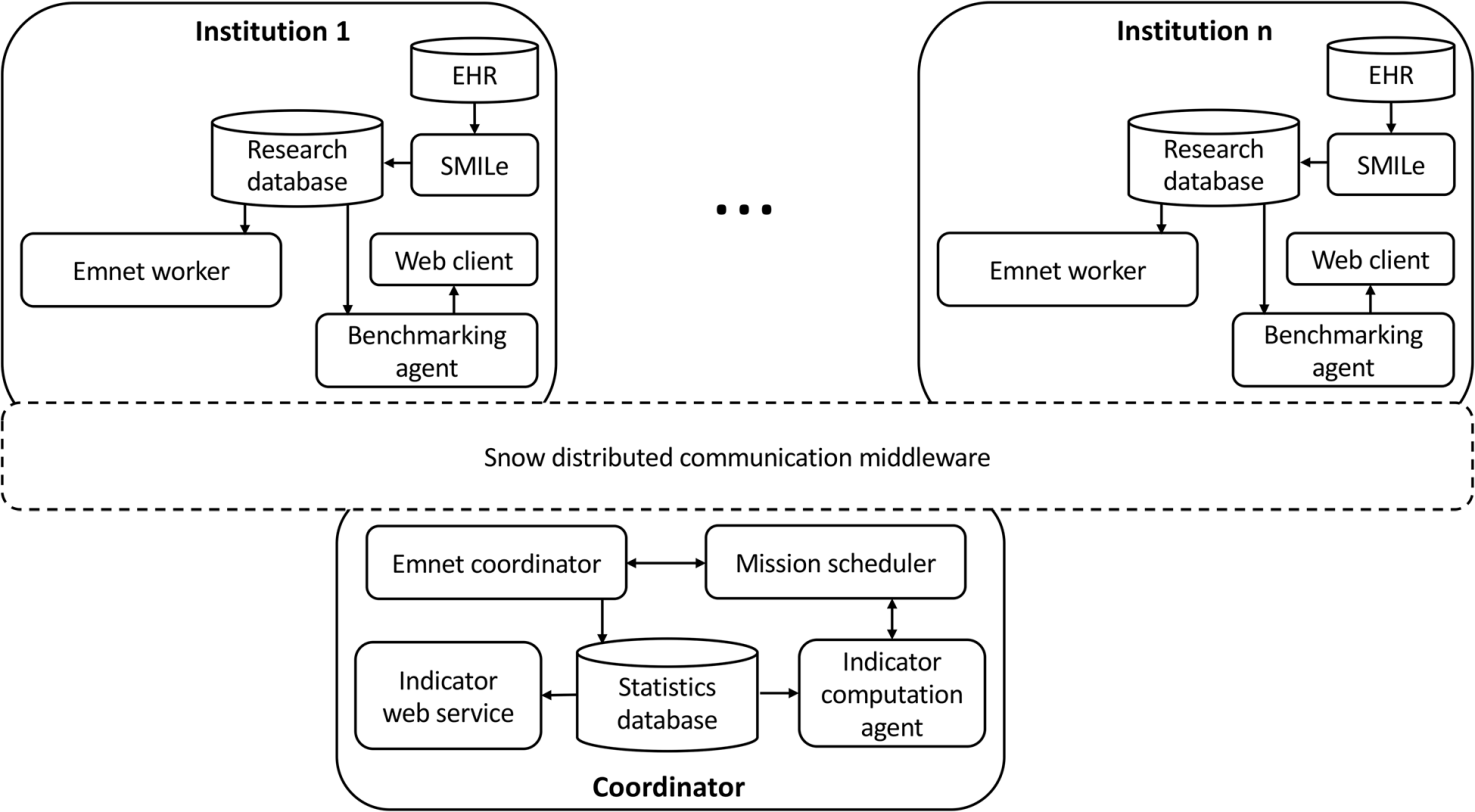
Privacy-preserving architecture for providing feedback to clinicians. Arrows indicate directions of information flow

The proposed privacy-preserving architecture (Fig. 1) contains a set of software components deployed on a server running at healthcare institutions and the coordinator. Secure communication between software components running at multiple entities were supported using the Snow system [39], which is a message-oriented middleware. The middleware has communication modules running at healthcare institutions and the coordinator. The communication module running at each healthcare institution establishes a secure connection with the module running at the coordinator, and all messages between healthcare institutions are relayed through the coordinator using end-to-end encryption. The design of the communication middleware satisfies the requirements of the Norwegian code of conduct for information security in the healthcare service sector where all communications with a healthcare institution should be initiated by the healthcare institution [40].

**Fig. 2:**
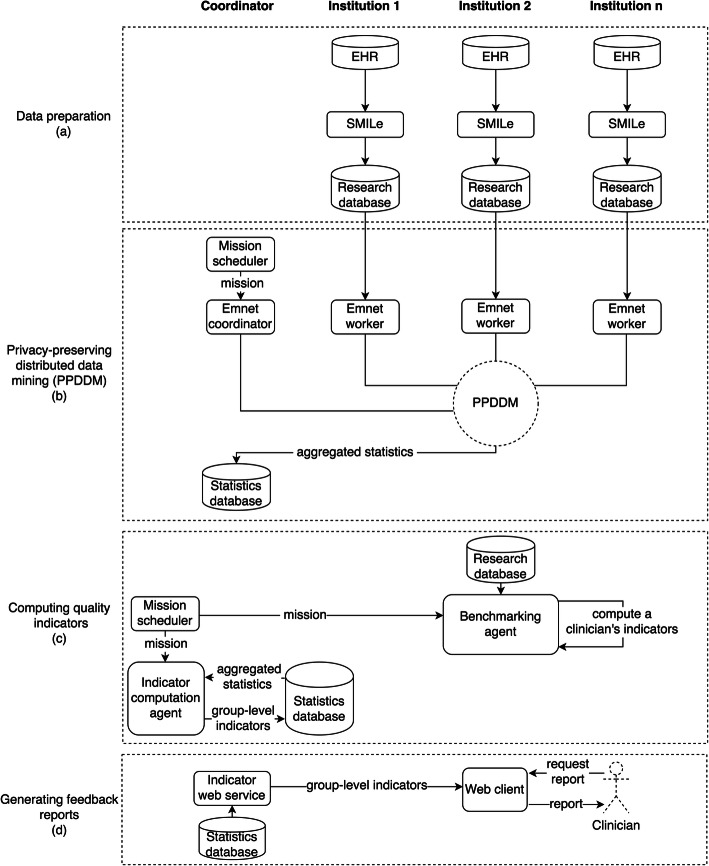
Data flow diagram for generating feedback reports

The main functionalities of the architecture are grouped into data preparation, scheduling the computation of quality indicators, computing quality indicators, and generating feedback reports. Figure 2 describes the data flow through all the components of the architecture to support these functionalities. The following sections describe how the architecture supports these functionalities.

#### Data preparation

The data sources for generating feedback were electronic health records (EHRs) of the participating healthcare institutions, which were proprietary EHR systems. As a result, the EHR data of each healthcare institution had to be transformed into a common data model, a data structure that standardize EHR data across healthcare institutions, and it can be possible to run standardized programs. To that end, the architecture used the Snow Medrave Interaction Library Extension (SMILe), which was developed in collaboration with Medrave Software AB [41]. The SMILe running at a healthcare institution extracts data daily from the local EHR system and transforms and loads the data into the research database. The research database has a common data model, which we have defined in an SQL format [42]. As shown in Fig. 2a, both the original EHR data and data transformed into the common data format remain at a healthcare institution.

The data transformation of SMILe includes pseudonymization, such as removing names, replacing national personal numbers and health personnel numbers with hash values, and replacing birthdates with birth years. The SMILe currently supports data extraction from three Norwegian primary care EHR systems that are the most widely used. Diagnoses and medical prescriptions in the EHRs and our research databases were classified according to the *International Classification of Primary Care, Second Edition* (ICPC-2)^1^ and the *Anatomical Therapeutic Chemical (ATC)*^2^*Classification System*, respectively.

#### Scheduling feedback

The architecture provides support for scheduling computations, such as computing quality indicators and generating feedback to clinicians, which is done by storing computation specifications at the coordinator. At the specified time, the *mission scheduler* executes a scheduled computation, denoted as a mission, by sending the mission specification to the appropriate software component (i.e., *the indicator computation agent* or *benchmarking agent*). The mission scheduler receives the mission status from the responsible components and maintains an overview of the overall computations in the system.

#### Computing quality indicators

The benchmarking agent (Fig. 2c) running at each healthcare institution computes the quality indicators of a clinician against the research database of the institution. The quality indicators are stored within the institution and cannot be revealed to anyone except the clinician to protect privacy. Therefore, the quality indicators are stored with a clinician pseudonym that is a secure hash of the health personnel number of the clinician.

Quality indicators of a group of peers across healthcare institutions (e.g., GP practices) are computed against distributed research databases. To that end, the architecture uses a PPDDM tool, called Emnet^3^ [37, 38]. Emnet is secure against semi-honest adversaries and supports the computation of various statistics. As shown in Fig. 2b, the tool consists of the Emnet coordinator and workers components that run at the coordinator and healthcare institutions, respectively. The *Emnet coordinator* receives queries (i.e., dataset creation and statistical computation) and broadcasts to *Emnet workers*. Each Emnet worker executes a dataset creation query against the local research database and locally stores the query results. The Emnet coordinator and workers jointly execute statistics queries on datasets distributed across healthcare institutions while protecting privacy. Emnet coordinator stores statistics results in the statistics database running at the coordinator.

A quality indicator of a group of peers is computed in two steps (Fig. 2b and c). First, Emnet [37] computes aggregated statistics on data distributed across healthcare institutions and stores the results in the statistics database, which is located at the coordinator. Second, the *indicator computation agent* computes indicators based on the results of Emnet and stores the results in the statistics database.

The percentage of respiratory tract infections (RTIs) cases treated with an antibacterial for systemic use (ATC = J01) is computed as follows. Emnet computes the number of cases diagnosed with RTIs (#cases) and the number of cases in which antibiotics were prescribed (#prescriptions). Then, the indicator computation agent computes the indicator based on the results of Emnet, (#prescriptions/ # *cases*) ∗ 100.

#### Generating feedback reports

After the benchmarking agent computes the performance indicators for a clinician, it sends a notification email regarding the availability of the feedback report to the clinician. As shown in Fig. 2d, clinicians access their feedback reports via a web client. Feedback reports of the currently logged-in clinician are generated on demand to restrict access. Feedback reports are generated based on a report template using a clinician’s indicators and the aggregated quality indicators of their peers, which are retrieved from the statistics database through the *indicator web service*. Only the respective clinician can view feedback reports and download as pdf files.

Feedback reports contain tables and graphs that enable the comparison of clinicians’ quality indicators with the aggregated quality indicators of their peers over time. A report also contains background information, such as a description of the peers and information about the relevant guidelines. We provide a sample feedback report on antibiotic prescriptions for an anonymous GP^4^ in Additional file 2.

### Implementation

The implementation of Emnet (implemented in Java) was outside the scope of the current paper, except for minor improvements. The other software components were implemented in Java. A MySQL database was used for the research database and statistics database. In addition, Hibernate was used to map the object-oriented data model to the relational database. The indicator web service was a restful web service implemented using Spring. Extensible Markup Language (XML) was used for specifying missions, and JavaScript Object Notation (JSON) was employed to specify the Emnet messages. The Snow system [39], a middleware for secure communication and coordination among involved entities, was implemented outside the scope of the current paper using the Extensible Messaging and Presence Protocol (XMPP) [43].

### Study design

#### Setup

We deployed the proposed architecture across three GP offices in Norway, and the *coordinator* was deployed at the University Hospital of North Norway. The number of GPs in the three GP offices varied over time, and 21 GPs agreed to receive a feedback report. The GPs had a total of 20,245 patients on their lists. The number of registered patients also varied during the study period because patients change their regular GPs from time to time.

The *coordinator* was deployed on an Intel Xeon X3220 2.4 GHz quad-core processor with 8 GB of RAM with Ubuntu 14.04. The software components at the two of the GP offices were deployed on an Intel i5-5300U 2.3 GHz dual-core processor with 8 GB of RAM with Windows Server 2012 R2. The software components at the third GP office were deployed on an Intel i5-5300U 2.3 GHz dual-core processor with 16 GB of RAM with Windows Server 2012 R2.

#### Antibiotic prescription feedback for clinicians

We applied the proposed architecture for generating feedback on the antibiotic prescriptions by the GPs for RTIs for which an antibiotic is generally not recommended. The selected RTIs are consultations for acute upper respiratory infection (ICPC-2e = R74), acute sinusitis (ICPC-2e = R75), acute laryngitis/tracheitis (ICPC-2e = R77), acute otitis media/myringitis (ICPC-2e = H71), and unspecified respiratory infections (ICPC-2e = R83). Acute bronchitis (ICPC-2e = R78) was also included because it is most often a viral infection, and the use of an antibiotic is rarely recommended.

Each GP received one feedback report in 2019 that covered the GP’s antibiotic prescriptions for the selected RTIs combined and each of the diagnoses separately between 2015 and 2018. All of the statistics in the report were stratified by year and antimicrobial spectrum (broad- and narrow-spectrum antibiotics). Narrow-spectrum antibiotics include fenoxymetylpenicillin (J01CE), and broad-spectrum antibiotics include tetracyclinces (J01A), broad spectrum penicillines (J01C except J01CE), cephalosporins (J01D), trimetoprim and sulfonamides (J01E), macrolides (J01F), and quinolones (J01M).

The feedback reports contain tables that present the number of cases diagnosed. The reports also contain the following indicators (based on the indicators proposed by the European surveillance of antimicrobial consumption [34]) for all the selected RTIs combined and each of the diagnoses separately:

- The percentage of diagnosed cases treated with an antibacterial for systemic use;
- The percentage of cases treated with narrow-spectrum antibiotics among all diagnosed cases treated with antibiotics; and
- The percentage of cases treated with broad-spectrum antibiotics among all diagnosed cases treated with antibiotics.

We refer interested readers to Additional file 1 for more information about the indicators computed in the current study. The indicators were presented as a time series graph comparing the performance indicators of a GP with the average performance indicators of all participating GPs. In this study, each GP received a single feedback report as an encrypted pdf file through email, and a decryption key was sent through the GP’s phone.

#### Monitoring group-level antibiotic prescriptions

The architecture is intended to enable monitoring of the quality of care (e.g., antibiotic prescriptions) at the group level. Measuring the effects of interventions involves computing indicators for the intervention group, before and after receiving feedback and for the control group. The current study only assessed antibiotic prescription information before the GPs received feedback. We computed the percentage of antibiotic prescriptions of the three GP offices for the selected RTIs combined. We also calculated the percentages of broad- and narrow-spectrum antibiotic prescriptions. Then, we computed the percentage of antibiotic prescriptions for each of the diagnoses separately. All the statistics were yearly stratified.

#### Computation time

We evaluated the efficiency of the architecture by measuring the time the architecture took to generate feedback reports. We ran each computation five times and reported the average computation time. We measured both the total and sub-computation times.

#### Ethics

The regional ethics committee of north Norway stated that a quality improvement study does not require ethical approval [44]. In addition, the study was exempt from seeking ethical approval because the objective of the antibiotic study was to evaluate the feasibility of our architecture in practice. Although the General Data Protection Regulation (GDPR) is incorporated into Norwegian law, the Norwegian patient record law is still applicable [16] because the GDPR allows possibility to introduce more specific national rules [45]. The Norwegian patient record law exempts patient consent for quality improvement purposes. Therefore, our study did not require ethical approval and consent from patients. However, we have received approvals from the GPs.

## Results

### Security analysis

The architecture computes group-level indicators of peers across healthcare institutions using Emnet [37], which does not reveal sensitive information of patients, clinicians, healthcare institutions. The performance indicators of a clinician are computed within a healthcare institution, and access to feedback reports for a clinician is protected with secure authentication. Therefore, the architecture satisfies both privacy requirements 1 and 2. The architecture reveals statistics generated from the combined data of multiple healthcare institutions, which are not considered sensitive information. The architecture also satisfies requirement 3 and 4, since Emnet is secure against semi-honest adversary [37].

### Antibiotic prescription feedback for clinicians

Feedback reports were provided to 21 GPs in 2019. The feedback provided to the GPs was a single pdf report on their antibiotic prescriptions for treating selected RTIs between 2015 and 2018. The indicators were stratified by year, diagnoses, and antibiotic spectrum. We refer interested readers to a sample feedback report of an anonymous GP provided as Additional file 2.

**Fig. 3:**
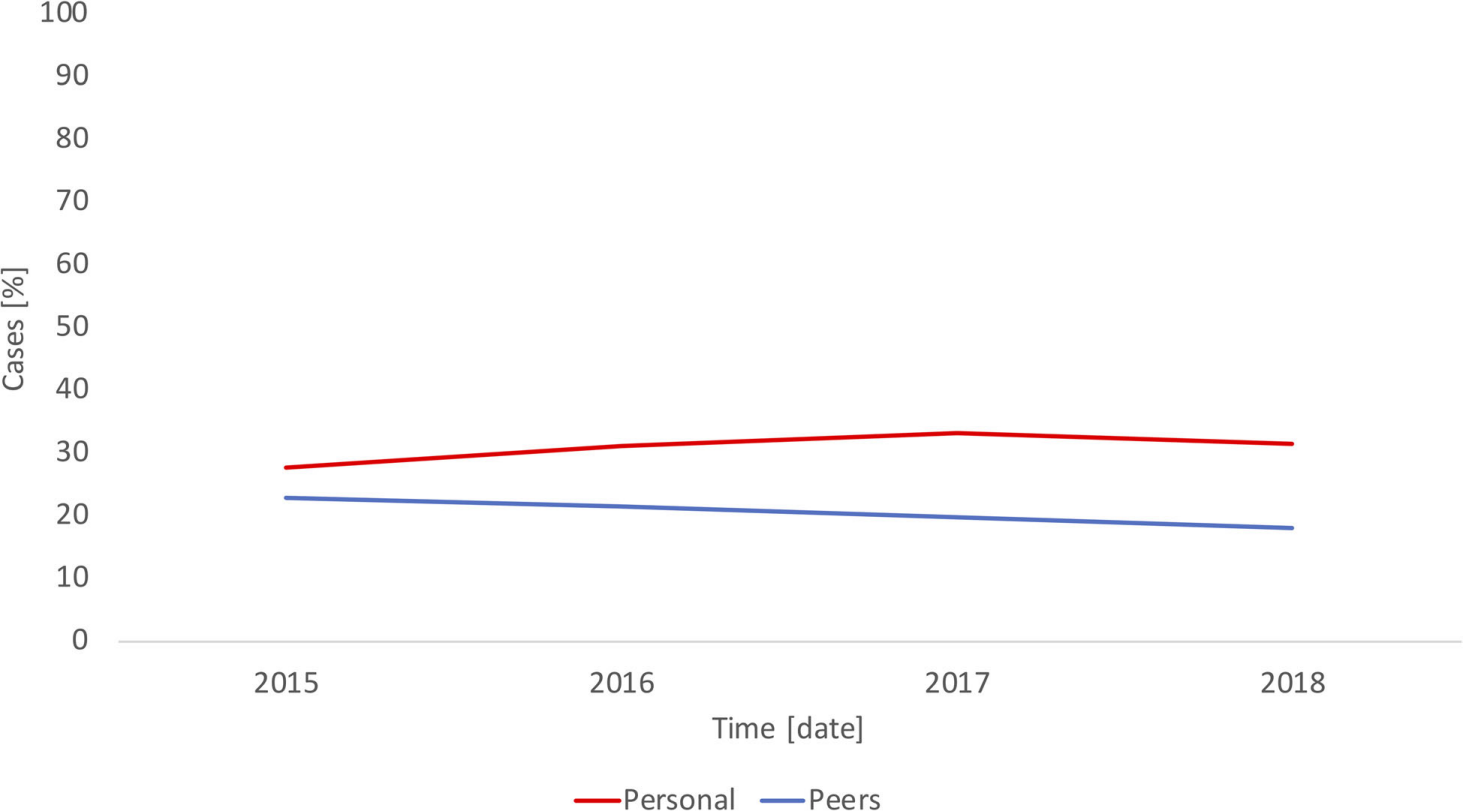
Antibiotic prescriptions for respiratory tract infections by an anonymous general practitioner in comparison to peers

Figure 3 shows the antibiotic prescriptions of an anonymous GP for the selected RTIs in comparison to his or her peers, which is extracted from the pdf report provided to the GP. The changes in antibiotic prescribing habits shown in the figure were not caused by our feedback reports because the changes occurred before the feedback reports were provided to the GPs.

### Monitoring group-level antibiotic prescriptions

This section reports changes in the antibiotic prescriptions of a group of three GP offices between 2015 and 2018 (Table 1), which occurred before the GPs received feedback reports generated in the current study. Overall, there were 14,396 cases of the selected RTIs and 6200 unique patients. Of these cases, 2924 (20.3%) cases were treated with an antibiotic, and 40.8% (1194) of the antibiotic prescriptions were broad-spectrum antibiotics.

**Table 1:** Antibiotic prescriptions for respiratory tract infections

Year	Number of RTIs^a^ cases	Number of antibiotic prescriptions (%)	Number of narrow-spectrum antibiotic^b^ prescriptions (%)	Number of broad-spectrum antibiotic^c^ prescriptions (%)
2015	3121	713 (22.9)	361 (50.6)	352 (49.4)
2016	3339	722 (21.6)	412 (57.1)	310 (42.9)
2017	4034	790 (19.6)	481 (60.9)	309 (39.1)
2018	3902	699 (17.9)	476 (68.1)	223 (31.9)

^a^ Respiratory tract infections (RTIs) include ICPC-2e = R74, R75, R77, R78, R83, and H71

^b^ Narrow-spectrum antibiotics include ATC = J01CE

^c^ Broad-spectrum antibiotics include ATC = J01A, J01C (except J01CE), J01D, J01E, J01F, J01M, and J01XX05

Antibiotic prescriptions decreased from 22.9% in 2015 to 17.9% in 2018 (absolute difference, 5%), and broad-spectrum antibiotic prescriptions decreased from 49.4 to 31.9% (absolute difference, 17.5%) during the study period.

**Fig. 4:**
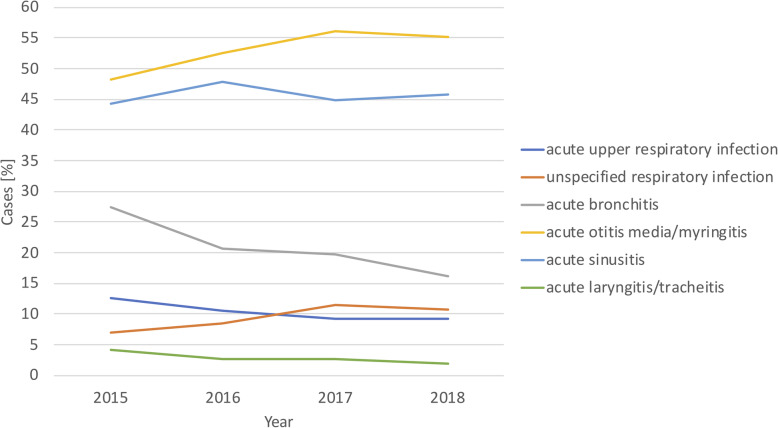
Antibiotic prescriptions for respiratory tract infections across three Norwegian general-practitioner offices

Figure 4 shows the antibiotic prescriptions stratified by the RTIs selected in the study. Antibiotic prescriptions for acute upper respiratory infections decreased from 12.60% in 2015 to 9.31% in 2018 (absolute difference, 3.28%) and acute bronchitis decreased from 27.33 to 16.10% (absolute difference, 11.23%). In contrast, antibiotic prescriptions for acute sinusitis increased from 44.30 to 45.84% (absolute difference, 1.54%) and acute otitis media/myringitis increased from 48.15 to 55.19% (absolute difference, 7.05%).

### Computation time

Generating feedback reports involve multiple rounds, such as the dataset creation where each GP office executes the query and locally stores the results, the statistical computation of the distributed data, and the generation of the feedback reports. The system took 13.3 s to execute a dataset creation query for the selected RTIs across the three GP offices. It took 13.7 s and 20.5 s to compute the number of RTIs cases and the cases treated with an antibiotic, respectively. In total, the system took 49.03 s to calculate the percentage, stratified by year, of RTI cases treated with an antibiotic. The benchmarking agent took 14 s to compute all of the indicators stratified by year for a GP. The generation of a pdf report based on the indicators of the GP and the aggregated indicators of peers took 15 s.

## Discussion

### Privacy-preserving architecture

The paper proposed a privacy-preserving architecture that enables clinicians to compare their clinical performance with peers across multiple healthcare institutions, and to monitor the quality of care at the group level.

De-identification is a common approach to protecting privacy during centralized data collection. De-identification requires a balance between data utility and privacy where strong privacy protection necessitates significant alterations to the data that considerably lower the data utility [46]. For example, an address may need to be generalized to a higher geographic location to protect privacy, but it limits possible stratifications of the data analysis. In contrast, our solution protects privacy without compromising data utility. It is also possible to infer private information about the institution from a de-identified data, which may not be acceptable by the institution [26], whereas our architecture also protects the privacy of the healthcare institutions.

The privacy-preserving distributed computation provided by Emnet [37] is efficient and scalable because the data sources locally perform record-level computations in parallel, and distributed secure computations are only executed to combine the local results. We found that computing the number of patients diagnosed with RTIs and treated with antibiotics across three GP offices took 13.7 s and 20.5 s, respectively. The computation time is acceptable, especially for scheduled computations where the results are not expected immediately.

The proposed architecture does not require extractions of data outside healthcare institutions. As a result, healthcare institutions maintain physical access control concerning deciding who can access a specific dataset, when the dataset can be accessed, what analysis can be performed on the dataset, and so on. Healthcare institutions may have different local data-access requirements [47]. Thus, the requirements for data processing should be at least as restrictive as all the individual healthcare institutions.

The architecture currently supports data-access control based on manual reviews of data analysis requests, which is similar to the approach used in several distributed health data reuse networks [48]. Existing techniques allow data controllers (i.e., healthcare institutions) to define their data-access requirements that enable enforcing automated access control [49, 50].

### Privacy-preserving feedback on clinical performance

The architecture was implemented and deployed in three Norwegian GP offices, and it was applied to provide feedback to GPs on their antibiotic prescriptions for the selected RTIs. We provided a single feedback report to 21 GPs.

Studies have shown the positive effects of feedback that compares a clinician’s performance with peers for behavioral changes in healthcare providers [5, 7, 8]. However, existing studies have been based on a centralized collection of data from participating healthcare institutions, which may raise privacy concerns. In addition, the outcomes of the study interventions might have been influenced by the fact that the subjects were being observed. However, our architecture provides feedback while protecting the privacy of clinicians, which may minimize such biases.

The usefulness of comparison with peers depends on the representativeness of the participating peers. Studies have shown that healthcare providers are often reluctant to share their data when they have privacy concerns [17]. Therefore, providing privacy protection may increase the willingness of healthcare providers to participate and, consequently, improve the representativeness of participating healthcare providers and the relevance of comparison with peers.

### Privacy-preserving monitoring of the quality of care

The architecture enables monitoring of the absolute difference in quality indicators for a particular group between two time points, which allows measuring the change in quality indicators before and after an intervention. We applied the architecture to measure changes in the antibiotic prescriptions of three GP offices between the year 2015 and 2018. Health authorities and policymakers can use the architecture to monitor the quality of care and the effects of education or regulatory interventions over time.

Studies evaluating the effects of interventions have been based on the centralized collection of data from all participating institutions, which is the same way they generated feedback to the clinicians [5, 7, 8]. In contrast, our architecture does not centrally collect data, and it protects privacy. However, our architecture must be extended with support for more statistics. For example, privacy-preserving distributed statistical tests are required to determine whether there are any statistically significant differences between the intervention and control groups.

### Limitations

Several studies have shown that the comparison with peers has a positive effect on behavioral change [4–8]. Whether regression towards the mean of peers is good, however, depends on the excellence of the peers’ practice, which may not always be taken for granted. Nonetheless, healthcare providers have legitimate privacy concerns [17]. However, the perception of healthcare providers toward our solution and its effect on their behavioral changes was not evaluated.

We assumed that the patient population served by a group of clinicians was homogenous. However, clinicians may often treat specific patient groups, for example, children and elderly individuals. To that end, we plan to extend the current implementation of the architecture to support the stratification of feedback reports based on such characteristics as age, sex, and health conditions.

We also assumed that all participating institutions are online at any given time, which held true for the three GP offices involved in our study. However, as the number of participating healthcare institutions increases, so does the number of offline institutions at any given time.

The other challenge is whether the PPDDM scales as the number of participating institutions increases by a large magnitude. For example, thousands of GP offices exist in Norway. We have proposed a technique that can scale Emnet to a very large number of data sources, but it is not yet implemented [38].

The architecture currently supports the generation of feedback reports that compare the clinical performance of a clinician with average peer performance. We plan to extend the architecture with a secure protocol for computing whether a clinician is a top-10% performer, which is known to influence behavioral change [5].

The proposed architecture has several benefits. However, its implementation, deployment, and management are more complex than solutions based on a centralized collection of data.

## Conclusions

We have demonstrated the feasibility of generating feedback to clinicians that enables them to compare their own performance with that of their peers while protecting privacy. Comparisons with peers are known to be efficient for quality improvement. The comparison with peers across multiple healthcare institutions is especially more important for smaller healthcare institutions, such as GP offices, where few peers within an institution are available to make a comparison. We also demonstrated the feasibility of monitoring the quality of services at a group level that enables evaluating the effects of education or regulatory interventions over time.

## Supplementary information

**Additional file 1.** It contains a descriptions of indicators for antibiotic prescriptions.

**Additional file 2.** It contains a sample feedback report of an anonymous general practitioner (GP). The file is obtained with a written permission of the GP.

## Data Availability

Datasets available at three GP offices were used for the study in this paper. A privacy-preserving distributed data mining technique was used for the data analysis without extracting data outside the organizations, and restrictions apply concerning making these data publicly available.

## Abbreviations

ATC: The Anatomical Therapeutic Chemical Classification System
EHR: Electronic health record
GDPR: General Data Protection Regulation
GP: General practitioner
ICPC: International Classification of Primary Care
PPDDM: Privacy-preserving distributed data mining
RTI: Respiratory tract infection
SMILe: Snow Medrave Interaction Library Extension
XMPP: Extensible Messaging and Presence Protocol

## Footnotes

1 The ICPC-2 code can be found at https://ehelse.no/kodeverk/icpc-2e%2D%2Denglish-version.

2 The ATC codes can be found at https://www.atccode.com/.

3 Emnet is an Amharic word for trust. Amharic is the official language of Ethiopia.

4 Feedback report of the anonymous GP was included with a written permission of the GP.
