# Supplementary material for: Privacy-preserving architecture for providing feedback to clinicians on their clinical performance
Source: BMC Med Inform Decis Mak. 2020 Jun 22;20:116. doi: 10.1186/s12911-020-01147-5 (PMC7310252; doi:10.1186/s12911-020-01147-5)
Supplement: Supplementary file 1 — Additional file 1. It contains a descriptions of indicators for antibiotic prescriptions. [file 12911_2020_1147_MOESM1_ESM.docx]

# Indicators for measuring antibiotic prescriptions

The standard measure for reporting of drug utilization is the number of defined daily dosage (DDD), which is defined as “the average maintenance dose of the drug when used on its major indication in adults.” Drug utilization is often presented as the number of DDDs per 1000 inhabitants per day (DID), which provides an estimate of the proportion of the diagnosed cases treated with the drug daily. For drugs normally used for short periods, it is often presented as the number of DDDs per inhabitant per year (DIY). DIY is interpreted as the average number of days each individual consumed the drug annually [1].

## General indicators

WHO developed a set of general indicators intended to measure the general prescribing tendencies independent of specific diagnoses [2]. The proposed indicators are average number of drugs per encounter, percentage of drugs prescribed by generic name, percentage of encounters with antibiotic prescribed, percentage of encounters with an injection prescribed, and percentage of drugs prescribed from essential drug list or formulary.

European Surveillance of Antimicrobial Consumption (ESAC) [3] developed twelve drug specific indicators for monitoring antibiotics use at GP practices. The first five indicators are the use of total antibiotic (J01^[[1]](#footnote-1)^) and four subclasses of antibiotics (i.e., J01C, J01D, J01F, J01M) measured in DID. The other four indicators are percentages of four smaller therapeutic groups (J01CE, J01CR, J01(DD+DE), J01MA) from the total antibiotics use measured in DID. The other indicator is the ratio of consumption of broad-spectrum (J01(CR+DC+DD+(F‐FA01))) and narrow-spectrum antibiotics (J01(CE+DB+FA01)) in number of DDDs or prescriptions. The other two indicators measure seasonal variation of total antibiotics and quinolone (J01M) consumption. Seasonal variation is measured as the ratio of antibiotics use in winter and summer.

Drug utilization 90% (DU90%) is a simple method for assessing the quality of drug prescription [4]. It is computed as the number of drugs accounted for 90% of the total volume of DDDs or number of prescriptions. Adherence to local or national prescription guidelines is evaluated with the proportion of prescribed drugs in the DU90% that appear on the list of drugs recommended in the guideline.

Table 1 summarizes the general indicators for antibiotic prescriptions. The indicators discussed so far are mainly focused on determining the overall prescribing performance. It is usually necessary to undertake health problem and demography specific investigations. Therefore, these indicators can be stratified with age, sex, symptoms, and/or diagnoses to understand prescription differences between different patient groups. However, the relevance of the stratified indicators for prescribers and policy makers may need to be formally assessed.

Table 1. General indicators for antibiotic prescriptions

| **Indicator** | **Description** | **Label** |
| --- | --- | --- |
| Number of DDDs per 1000 inhabitants per day | The number of DDDs per day divided by the denominator population multiplied by 1000 | DID |
| Number of DDDs per inhabitant per year | The number of DDDs per year divided by the denominator population | DIY |
| Number of prescriptions per 1000 inhabitants per year | The number of antibiotic prescriptions divided by the denominator population multiplied by 1000 | PIY |
| DU90% | The number of antibiotics accounted for 90% of the total number of DDDs or prescriptions | DU90% |
| Average number of antibiotics per encounter | The number of antibiotic prescriptions divided by the total number of encounters | APE |
| Percentage of an antibiotics use from the total antibiotics use | The DID of the drug divided by the DID of all drugs and multiplied by 100 | PA |
| Percentage of encounters with antibiotics prescribed | The total number of encounters during which an antibiotics were prescribed divided by the total number of encounters and multiplied by 100 | PEA |
| Ratio of two groups of antibiotics | The ratio of the number of DDDs of group one and group two antibiotic | Ratio |

## Disease specific indicators

This section describes antibiotic prescription indicators proposed for specific diagnoses/diseases and antibiotics. The indicators are based on the general indicators discussed above. ESAC developed 21 disease specific indicators for outpatient antibiotics prescriptions [5] that were the basis for the indicators (Table 2) we computed in this paper.

Table 2. Disease specific indicators computed in the current paper.

| **Disease** | **Indicator** | **Description** |
| --- | --- | --- |
| RTIs^*^ | Percentage of RTIs cases treated with an antibacterial for systematic use (J01) | The number of RTIs cases treated with antibiotics divided by the number of cases and multiplied by 100 |
|  | Percentage of cases treated with J01CE among all cases treated with J01 | The number of RTIs cases treated with J01CE divided by the number of cases treated with any antibiotic and multiplied by 100 |
|  | Percentage of cases treated with broad-spectrum antibiotics^**^ among all cases treated with J01 | The number of RTIs cases treated with broad-spectrum antibiotics divided by the number of cases treated with any antibiotic and multiplied by 100 |
| Acute bronchitis | Percentage of acute bronchitis cases treated with J01 | The number of acute bronchitis cases treated with antibiotics divided by the number of cases and multiplied by 100 |
|  | Percentage of acute bronchitis patients treated with J01CE among all cases treated with J01 | The number of acute bronchitis cases treated with J01CE divided by the number of cases treated with any antibiotic and multiplied by 100 |
|  | Percentage of acute bronchitis cases treated with broad-spectrum antibiotics among all cases treated with J01 | The number of acute bronchitis cases treated with broad-spectrum antibiotics divided by the number of cases treated with any antibiotic and multiplied by 100 |
| Acute upper respiratory infection | Percentage of acute upper respiratory infection cases treated with J01 | The number of acute upper respiratory infection cases treated with antibiotics divided by the number of cases and multiplied by 100 |
|  | Percentage of acute upper respiratory infection cases treated with J01CE among all cases treated with J01 | The number of acute upper respiratory infection cases treated with J01CE divided by the number of cases treated with any antibiotic and multiplied by 100 |
|  | Percentage of cases treated with broad-spectrum antibiotics among all cases treated with J01 | The number of acute upper respiratory infection cases treated with broad-spectrum antibiotics divided by the number of cases treated with any antibiotic and multiplied by 100 |
| Acute  sinusitis | Percentage of acute sinusitis cases treated with J01 | The number of acute sinusitis cases treated with antibiotics divided by the number of cases and multiplied by 100 |
|  | Percentage of acute sinusitis cases treated with J01CE among all cases treated with J01 | The number of acute sinusitis cases treated with J01CE divided by the number of cases treated with any antibiotic and multiplied by 100 |
|  | Percentage of acute sinusitis cases treated with broad-spectrum antibiotics among all cases treated with J01 | The number of acute sinusitis cases treated with broad-spectrum antibiotics divided by the number of cases treated with any antibiotic and multiplied by 100 |
| Acute otitis  media/myringitis | Percentage of acute otitis  media cases treated with J01 | The number of acute otitis media cases treated with antibiotics divided by the number of cases and multiplied by 100 |
|  | Percentage of acute sinusitis cases treated with J01CE among all cases treated with J01 | The number of acute otitis media cases treated with J01CE divided by the number of cases treated with any antibiotic and multiplied by 100 |
|  | Percentage of acute otitis media cases treated with broad-spectrum antibiotics among all cases treated with J01 | The number of acute otitis media cases treated with broad-spectrum antibiotics divided by the number of cases treated with any antibiotic and multiplied by 100 |
| Acute laryngitis/tracheitis | Percentage of acute laryngitis cases treated with J01 | The number of acute laryngitis cases treated with antibiotics divided by the number of cases and multiplied by 100 |
|  | Percentage of acute laryngitis cases treated with J01CE among all cases treated with J01 | The number of acute laryngitis cases treated with J01CE divided by the number of cases treated with any antibiotic and multiplied by 100 |
|  | Percentage of acute laryngitis cases treated with broad-spectrum antibiotics among all cases treated with J01 | The number of acute laryngitis cases treated with broad-spectrum antibiotics divided by the number of cases treated with any antibiotic and multiplied by 100 |
| Unspecified respiratory infection | Percentage of unspecified respiratory infection cases treated with J01 | The number of acute laryngitis cases treated with antibiotics divided by the number of cases and multiplied by 100 |
|  | Percentage of unspecified respiratory infection cases treated with J01CE among all cases treated with J01 | The number of unspecified respiratory infection cases treated with J01CE divided by the number of cases treated with any antibiotic and multiplied by 100 |
|  | Percentage of unspecified respiratory infection cases treated with broad-spectrum antibiotics among all cases treated with J01 | The number of unspecified respiratory infection cases treated with broad-spectrum antibiotics divided by the number of cases treated with any antibiotic and multiplied by 100 |

Norwegian government proposed a set of goals for evaluating the success of its aim towards 30% reduction of antibiotics use by 2020 compared to 2012 [6]. The goals include reducing total antibiotics use and antibiotics use for respiratory infections by 30 percent and 20 percent, respectively, measured in DID. The other measure is reduction of the number of prescriptions per 1000 inhabitants per year (PIY) to 250.

## Antibiotic specific indicators

Table 3 shows indicators for the usage of specific antibiotic.

Table 3. Indicators for the usage of specific antibiotic (Source: [3])

| **Antibiotics** | **Indicator** | **Label** |
| --- | --- | --- |
| J01 | DID | [J01_DID] |
|  | Ratio^+^ | [J01_SV] |
| J01C | DID | [J01C_DID] |
| J01D | DID | [J01D_DID] |
| J01F | DID | [J01F_DID] |
| J01M | DID | [J01M_DID] |
|  | Ratio^+^ | [J01M_SV] |
| J01CE | PA | [J01CE_%] |
| J01CR | PA | [J01CR_%] |
| J01(DD+DE) | PA | [J01DD+DE_%] |
| J01MA | PA | [J01MA_%] |
| J01(CR+DC+DD+(F‐FA01)) and J01(CE+DB+FA01) | Ratio | [J01_B/N] |

^+^ The ratio of total number of DDDs in winter quarters (October - December, January - March) and summer quarters (July – September and April - June) of a one-year period starting in July and ending in June the next calendar year.

# References

[1] WHO International Working Group for Drug, WHO Collaborating Centre for Drug Statistics Methodology, WHO International Working Group for Drug, Introduction to drug utilization research, World Health Organization, Oslo, 2003. http://www.who.int/medicines/areas/quality_safety/safety_efficacy/Drug%20utilization%20research.pdf?ua=1.

[2] World Health Organization, How to Investigate Drug Use in Health Facilities: Selected Drug Use Indicators, 1993.

[3] S. Coenen, M. Ferech, F.M. Haaijer‐Ruskamp, C.C. Butler, R.H.V. Stichele, T.J.M. Verheij, D.L. Monnet, P. Little, H. Goossens, European Surveillance of Antimicrobial Consumption (ESAC): quality indicators for outpatient antibiotic use in Europe, Qual. Saf. Health Care. 16 (2007) 440–445. https://doi.org/10.1136/qshc.2006.021121.

[4] U. Bergman, C. Popa, Y. Tomson, B. Wettermark, T.R. Einarson, H. Aberg, F. Sjöqvist, Drug utilization 90%--a simple method for assessing the quality of drug prescribing, Eur. J. Clin. Pharmacol. 54 (1998) 113–118.

[5] N. Adriaenssens, S. Coenen, Disease-specific antibiotic prescribing quality indicators report, University of Antwerp, Antwerp, 2010. https://ecdc.europa.eu/sites/portal/files/media/en/healthtopics/antimicrobial-resistance-and-consumption/antimicrobial-consumption/publications-documents/Documents/ESAC-Net-archive-report_disease_specific_antibiotic_prescribing_quality_indicators.pdf.

[6] Norwegian Ministries, National Strategy against Antibiotic Resistance 2015–2020, n.d. https://www.regjeringen.no/contentassets/5eaf66ac392143b3b2054aed90b85210/antibiotic-resistance-engelsk-lavopploslig-versjon-for-nett-10-09-15.pdf (accessed January 26, 2017).

1. All the antibiotics are denoted with ATC codes [↑](#footnote-ref-1)
