# Supplementary material for: Privacy-preserving architecture for providing feedback to clinicians on their clinical performance
Source: BMC Med Inform Decis Mak. 2020 Jun 22;20:116. doi: 10.1186/s12911-020-01147-5 (PMC7310252; doi:10.1186/s12911-020-01147-5)
Supplement: Supplementary file 2 — Additional file 2. It contains a sample feedback report of an anonymous general practitioner (GP). The file is obtained with a written permission of the GP. [file 12911_2020_1147_MOESM2_ESM.pdf]

Periodic feedback on your antibiotics prescriptions

Report period:
2015-01-01 - 2018-12-31

Learning healthcare system project
The Norwegian centre for e-health research
2019-08-21 12:40:54
Norway

1. Background

1.1. Objective

The report contains indicators for your antibiotics prescriptions patterns in comparison to the average of 20 general practitioners in three GP offices. The report is generated based on the clinical data collected in the EHRs of the three GP offices. The report aims to encourage rational antibiotics prescriptions and, consequently, minimize antibiotics resistance and adverse events.

1.2. Learning Healthcare System

This feedback report is generated by the learning healthcare system project at the Norwegian centre for e-health research. The project develops tools enabling continuous learning of the healthcare system with data collected in clinical practice and from patients. One of the results of the project is a privacy-preserving method for generating reports comparing the performance of a clinician to his/her peers across multiple health care providers.
Further information about the project and the method used to generate the report can be found from the project website
https://ehealthresearch.no/en/projects/learning-health-care-system-toolbox
If you have questions about the report contact us at
snow.support@ehealthresearch.no

1.3. Disclaimer

We recommend that the report should be interpreted in relation to the Norwegian guidelines for antibiotics use in primary care
http://www.antibiotikaiallmennpraksis.no/index.php.

2. Feedback

The current report contains feedback on antibiotics prescriptions for a selection of respiratory tract infections. The infections are chosen because it is usually not advised to treat them with antibiotics. The report presents the number of patients diagnosed with a particular diagnosis, what percentage of these patients were treated with antibiotics, and what percentages of the antibiotics were narrow spectrum and broad spectrum antibiotics. The report also contains the corresponding average of the participating general practitioners.

2.1. Respiratory tract infections

This section presents antibiotics consumption statistics for respiratory tract infections (i.e., ICPC2 = R74, R75, R77, R78, R83 and H71). Table 1 shows the number of your patients diagnosed with respiratory tract infections as well as the number of those treated with antibiotics, narrow spectrum antibiotics (i.e., ATC = J01CE) and broad spectrum antibiotics (i.e., ATC = J01A, J01C except J01CE, J01D, J01E, J01F or J01M).

Table 1: The number of your patients diagnosed with respiratory tract infections.

Time period		Patients diagnosed	Patients treated with antibiotics		
From	To		Total	Narrow spectrum	Broad spectrum
2015-01-01	2015-12-31	144	40	22	18
2016-01-01	2016-12-31	90	28	23	5
2017-01-01	2017-12-31	127	42	26	16
2018-01-01	2018-12-31	134	42	29	13

2.1.1. Total antibiotics prescriptions

Figure 1 shows the percentage of your patients treated with antibiotics among those who were diagnosed with respiratory tract infections.

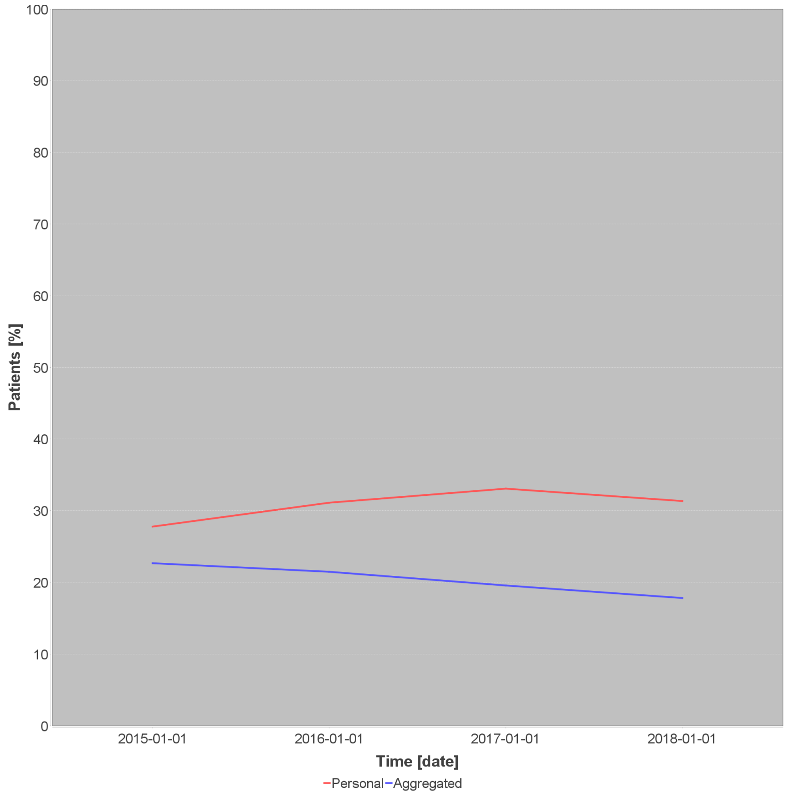

Figure 1: The percentage of patients diagnosed with respiratory tract infections treated with antibiotics.

2.1.2. Narrow spectrum antibiotics prescriptions

Figure 2 shows the percentage of respiratory tract infections patients treated with narrow spectrum antibiotics (i.e., ATC = J01CE) among those who were treated with antibiotics. A higher value indicates that a majority of the cases were treated with the recommended antibiotics. An increase in the prescriptions of narrow-spectrum antibiotics also indicates a decrease in the prescriptions of broad-spectrum antibiotics and vice versa.

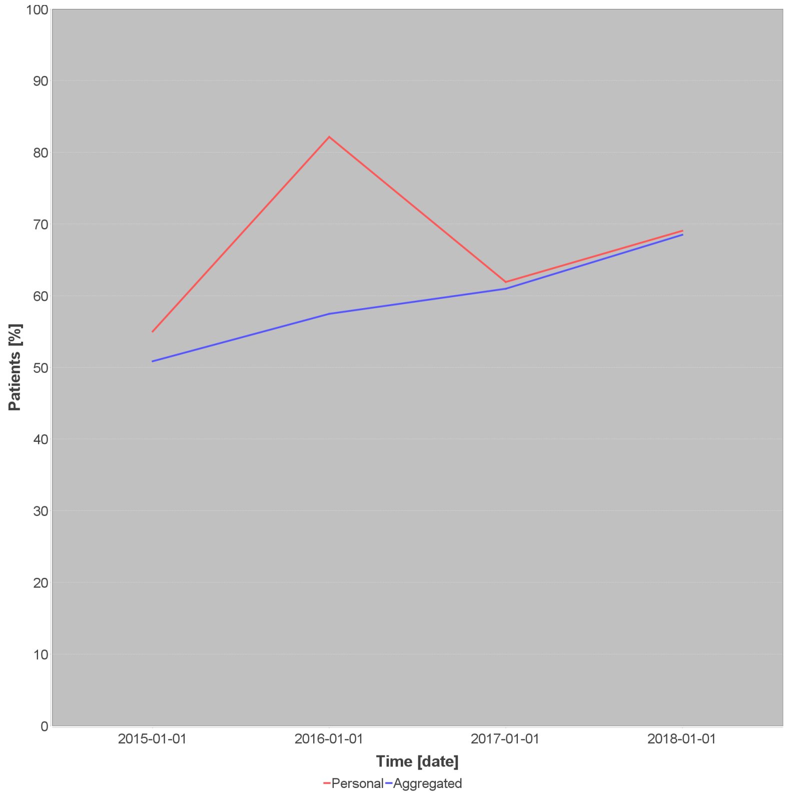

Figure 2: The percentage of narrow spectrum antibiotics (i.e., ATC = J01CE) of all antibiotics prescribed for respiratory tract infections patients.

2.2. Acute upper respiratory infection

This section presents antibiotics consumption statistics for acute upper respiratory infection (i.e., ICPC2 = R74). Table 2 shows the number of your patients diagnosed with acute upper respiratory infection as well as the number of those treated with antibiotics, narrow spectrum antibiotics (i.e., ATC = J01CE) and broad spectrum antibiotics (i.e., ATC = J01A, J01C except J01CE, J01D, J01E, J01F or J01M).

Table 2: The number of your patients diagnosed with acute upper respiratory infection.

Time period		Patients diagnosed	Patients treated with antibiotics		
From	To		Total	Narrow spectrum	Broad spectrum
2015-01-01	2015-12-31	23	1	0	1
2016-01-01	2016-12-31	16	4	4	0
2017-01-01	2017-12-31	39	5	4	1
2018-01-01	2018-12-31	21	2	2	0

2.2.1. Total antibiotics prescriptions

Figure 4 shows the percentage of your patients treated with antibiotics among those who were diagnosed with acute upper respiratory infection.

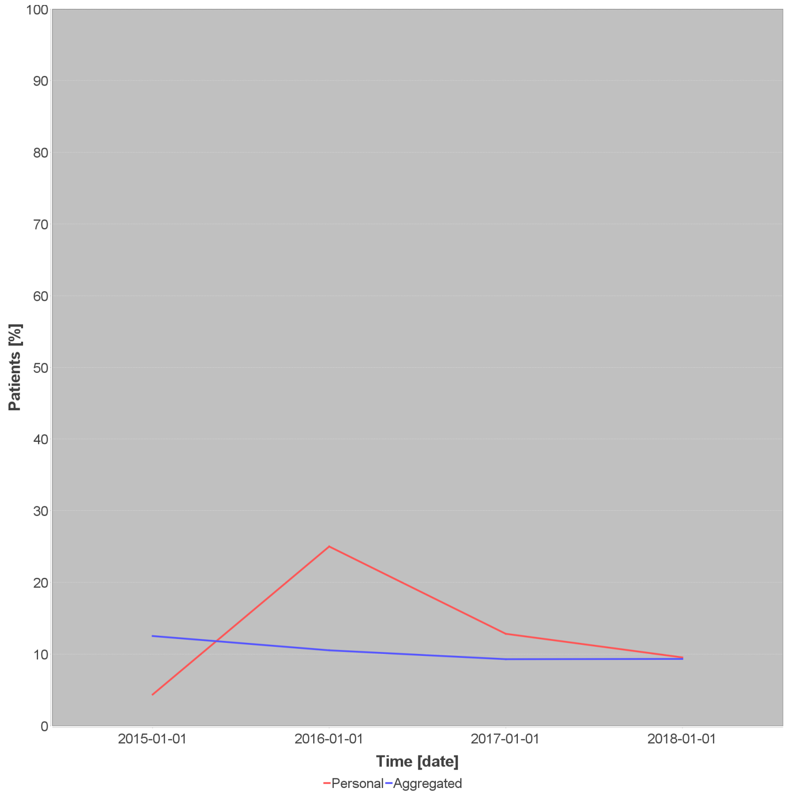

Figure 4: The percentage of patients diagnosed with acute upper respiratory infection treated with antibiotics.

2.2.2. Narrow spectrum antibiotics prescriptions

Figure 5 shows the percentage of acute upper respiratory infection patients treated with narrow spectrum antibiotics (i.e., ATC = J01CE) among those who were treated with antibiotics. A higher value indicates that a majority of the cases were treated with the recommended antibiotics. An increase in the prescriptions of narrow-spectrum antibiotics also indicates a decrease in the prescriptions of broad-spectrum antibiotics and vice versa.

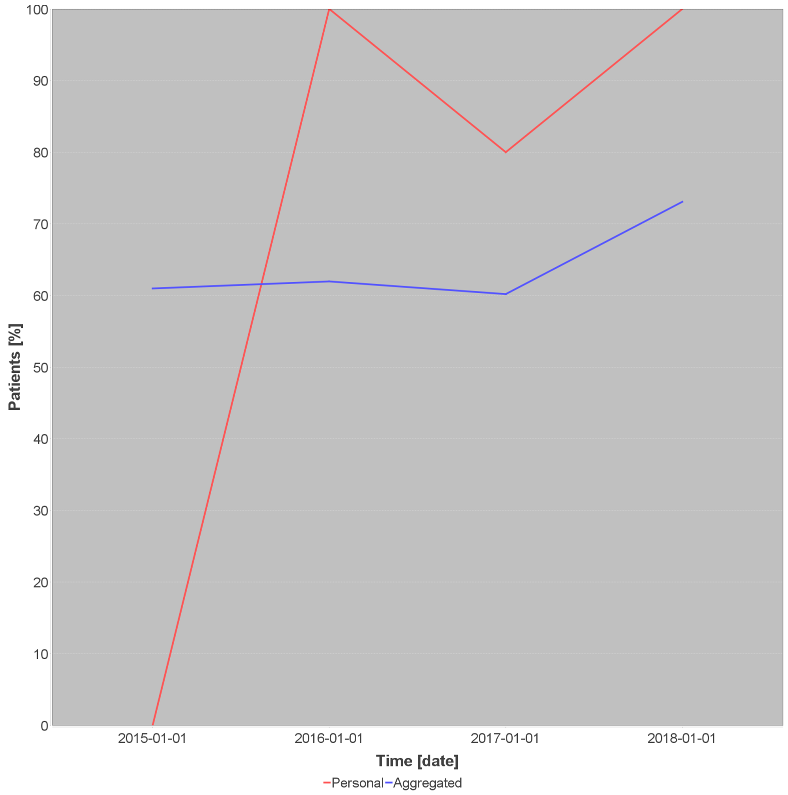

Figure 5: The percentage of narrow spectrum antibiotics (i.e., ATC = J01CE) of all antibiotics prescribed for acute upper respiratory infection patients.

2.3. Acute otitis media/myringitis

This section presents antibiotics consumption statistics for acute otitis media/myringitis (i.e., ICPC2 = H71). Table 3 shows the number of your patients diagnosed with acute otitis media/myringitis as well as the number of those treated with antibiotics, narrow spectrum antibiotics (i.e., ATC = J01CE) and broad spectrum antibiotics (i.e., ATC = J01A, J01C except J01CE, J01D, J01E, J01F or J01M).

Table 3: The number of your patients diagnosed with acute otitis media/myringitis.

Time period		Patients diagnosed	Patients treated with antibiotics		
From	To		Total	Narrow spectrum	Broad spectrum
2015-01-01	2015-12-31	10	4	4	0
2016-01-01	2016-12-31	9	6	6	0
2017-01-01	2017-12-31	23	12	8	4
2018-01-01	2018-12-31	17	11	11	0

2.3.1. Total antibiotics prescriptions

Figure 7 shows the percentage of your patients treated with antibiotics among those who were diagnosed with acute otitis media/myringitis.

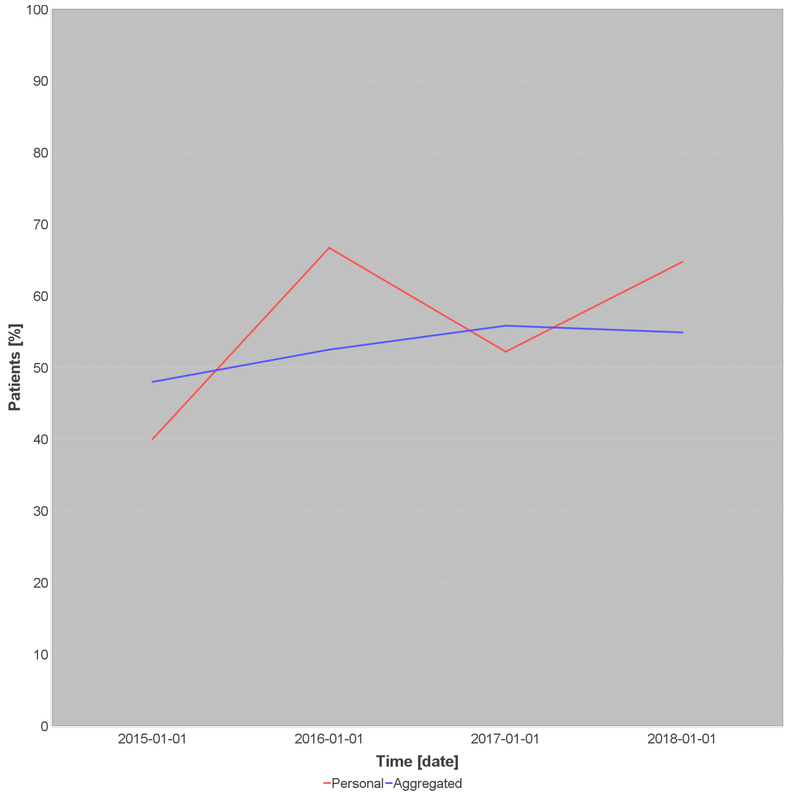

Figure 7: The percentage of patients diagnosed with acute otitis media/myringitis treated with antibiotics.

2.3.2. Narrow spectrum antibiotics prescriptions

Figure 8 shows the percentage of acute otitis media/myringitis patients treated with narrow spectrum antibiotics (i.e., ATC = J01CE) among those who were treated with antibiotics. A higher value indicates that a majority of the cases were treated with the recommended antibiotics. An increase in the prescriptions of narrow-spectrum antibiotics also indicates a decrease in the prescriptions of broad-spectrum antibiotics and vice versa.

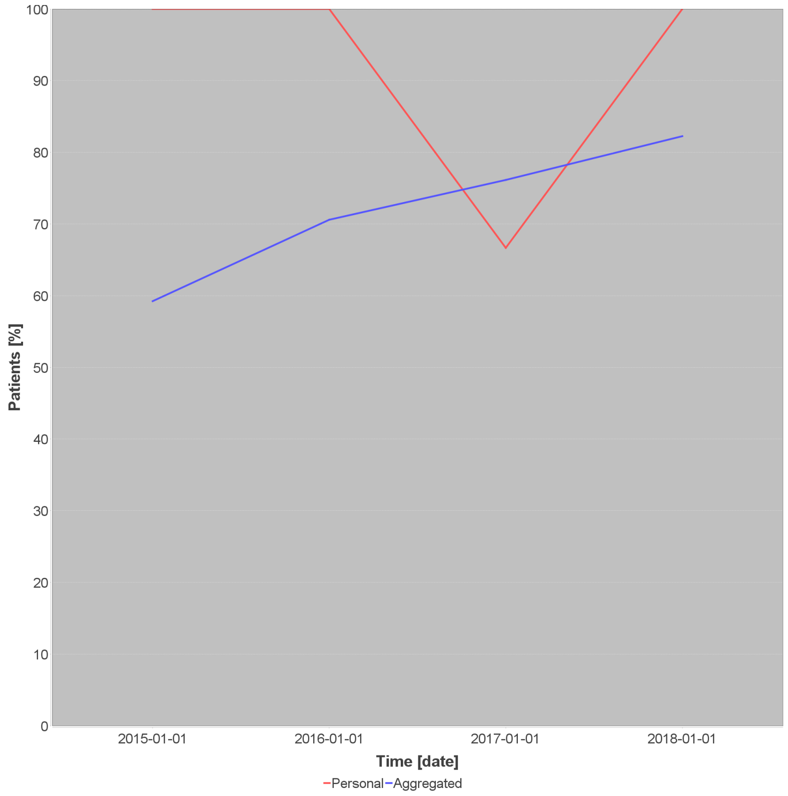

Figure 8: The percentage of narrow spectrum antibiotics (i.e., ATC = J01CE) of all antibiotics prescribed for acute otitis media/myringitis patients.

2.4. Acute bronchitis

This section presents antibiotics consumption statistics for acute bronchitis (i.e., ICPC2 = R78). Table 4 shows the number of your patients diagnosed with acute bronchitis as well as the number of those treated with antibiotics, narrow spectrum antibiotics (i.e., ATC = J01CE) and broad spectrum antibiotics (i.e., ATC = J01A, J01C except J01CE, J01D, J01E, J01F or J01M).

Table 4: The number of your patients diagnosed with acute bronchitis.

Time period		Patients diagnosed	Patients treated with antibiotics		
From	To		Total	Narrow spectrum	Broad spectrum
2015-01-01	2015-12-31	47	13	1	12
2016-01-01	2016-12-31	36	3	3	0
2017-01-01	2017-12-31	37	5	1	4
2018-01-01	2018-12-31	29	5	2	3

2.4.1. Total antibiotics prescriptions

Figure 10 shows the percentage of your patients treated with antibiotics among those who were diagnosed with acute bronchitis.

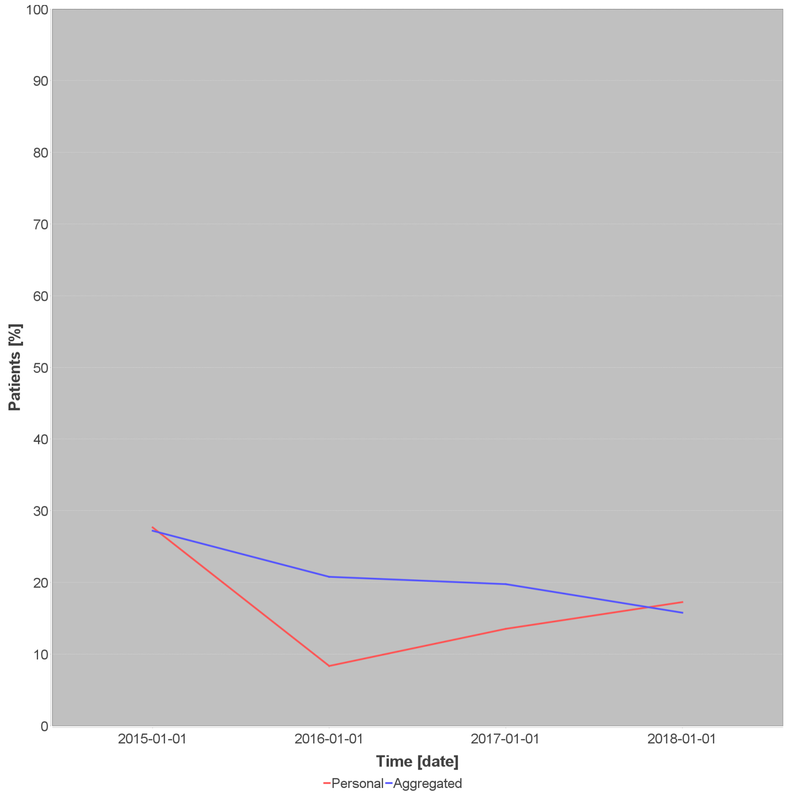

Figure 10: The percentage of patients diagnosed with acute bronchitis treated with antibiotics.

2.4.2. Narrow spectrum antibiotics prescriptions

Figure 11 shows the percentage of acute bronchitis patients treated with narrow spectrum antibiotics (i.e., ATC = J01CE) among those who were treated with antibiotics. A higher value indicates that a majority of the cases were treated with the recommended antibiotics. An increase in the prescriptions of narrow-spectrum antibiotics also indicates a decrease in the prescriptions of broad-spectrum antibiotics and vice versa.

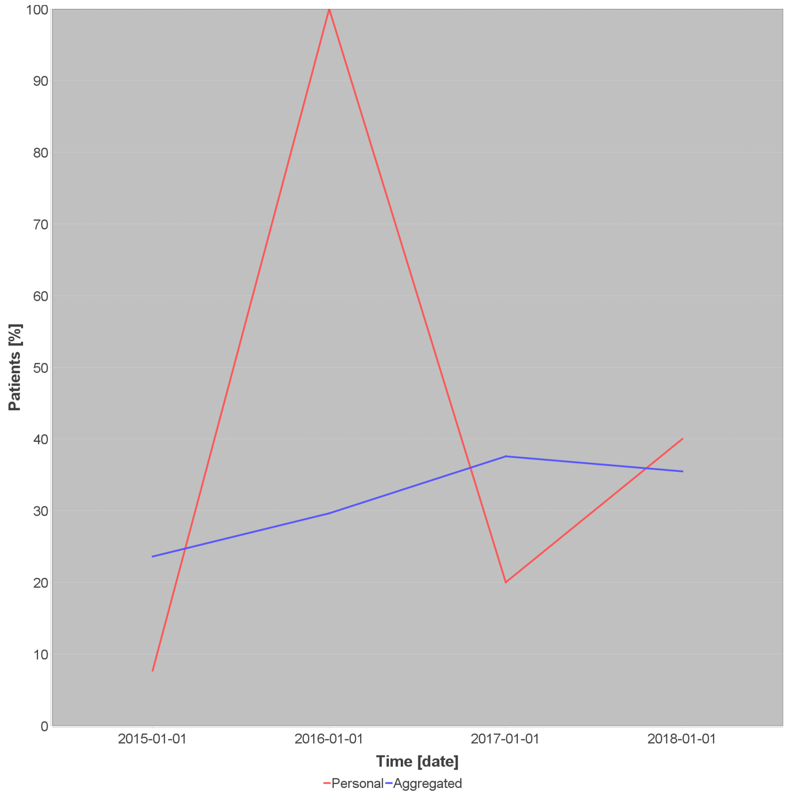

Figure 11: The percentage of narrow spectrum antibiotics (i.e., ATC = J01CE) of all antibiotics prescribed for acute bronchitis patients.

2.5. Acute sinusitis

This section presents antibiotics consumption statistics for acute sinusitis (i.e., ICPC2 = R75). Table 5 shows the number of your patients diagnosed with acute sinusitis as well as the number of those treated with antibiotics, narrow spectrum antibiotics (i.e., ATC = J01CE) and broad spectrum antibiotics (i.e., ATC = J01A, J01C except J01CE, J01D, J01E, J01F or J01M).

Table 5: The number of your patients diagnosed with acute sinusitis.

Time period		Patients diagnosed	Patients treated with antibiotics		
From	To		Total	Narrow spectrum	Broad spectrum
2015-01-01	2015-12-31	31	22	17	5
2016-01-01	2016-12-31	17	13	9	4
2017-01-01	2017-12-31	27	20	13	7
2018-01-01	2018-12-31	28	19	12	7

2.5.1. Total antibiotics prescriptions

Figure 13 shows the percentage of your patients treated with antibiotics among those who were diagnosed with acute sinusitis.

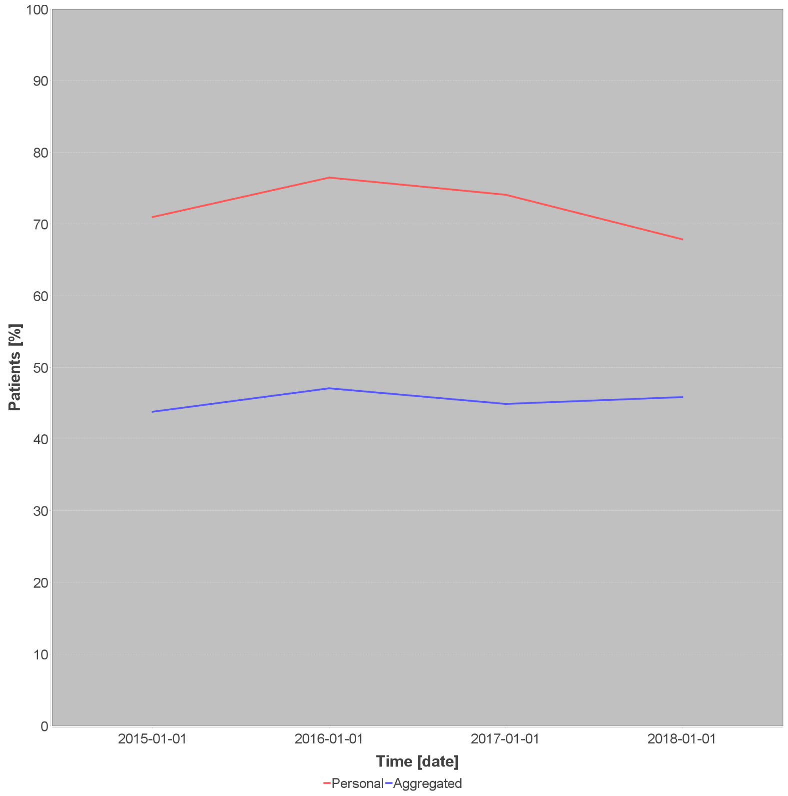

Figure 13: The percentage of patients diagnosed with acute sinusitis treated with antibiotics.

2.5.2. Narrow spectrum antibiotics prescriptions

Figure 14 shows the percentage of acute sinusitis patients treated with narrow spectrum antibiotics (i.e., ATC = J01CE) among those who were treated with antibiotics. A higher value indicates that a majority of the cases were treated with the recommended antibiotics. An increase in the prescriptions of narrow-spectrum antibiotics also indicates a decrease in the prescriptions of broad-spectrum antibiotics and vice versa.

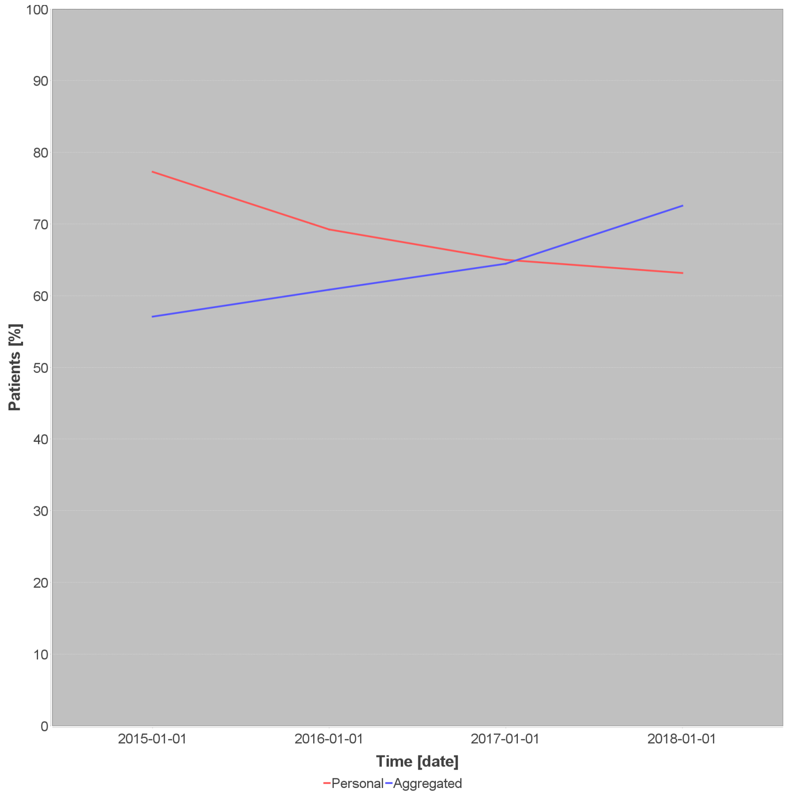

Figure 14: The percentage of narrow spectrum antibiotics (i.e., ATC = J01CE) of all antibiotics prescribed for acute sinusitis patients.

2.6. Acute laryngitis/tracheitis

This section presents antibiotics consumption statistics for acute laryngitis/tracheitis (i.e., ICPC2 = R77). Table 6 shows the number of your patients diagnosed with acute laryngitis/tracheitis as well as the number of those treated with antibiotics, narrow spectrum antibiotics (i.e., ATC = J01CE) and broad spectrum antibiotics (i.e., ATC = J01A, J01C except J01CE, J01D, J01E, J01F or J01M).

Table 6: The number of your patients diagnosed with acute laryngitis/tracheitis.

Time period		Patients diagnosed	Patients treated with antibiotics		
From	To		Total	Narrow spectrum	Broad spectrum
2015-01-01	2015-12-31	2	0	0	0
2016-01-01	2016-12-31	4	1	1	0
2017-01-01	2017-12-31	0	0	0	0
2018-01-01	2018-12-31	2	0	0	0

2.6.1. Total antibiotics prescriptions

Figure 16 shows the percentage of your patients treated with antibiotics among those who were diagnosed with acute laryngitis/tracheitis.

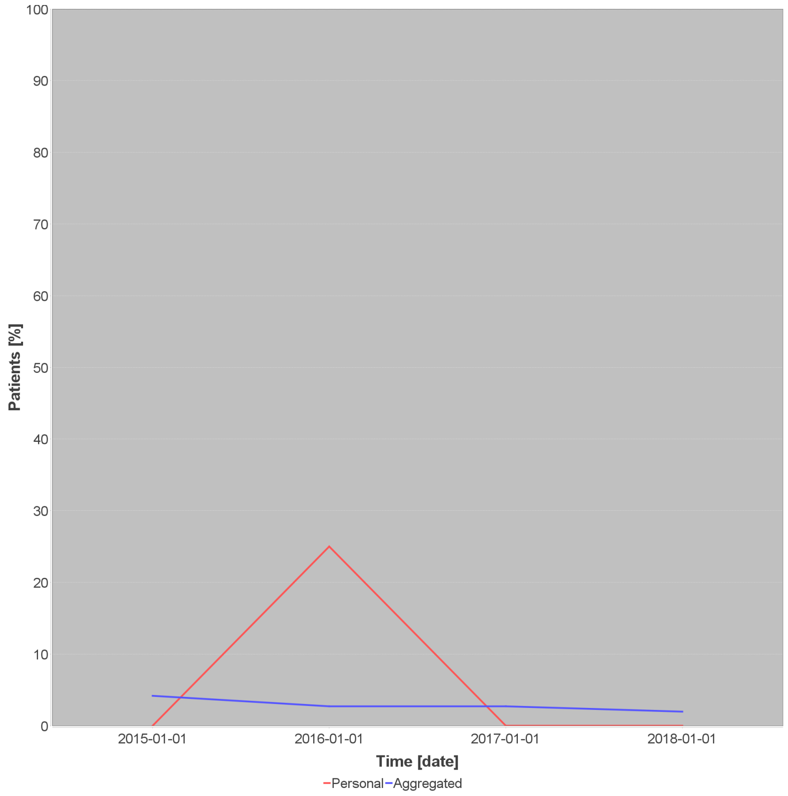

Figure 16: The percentage of patients diagnosed with acute laryngitis/tracheitis treated with antibiotics.

2.6.2. Narrow spectrum antibiotics prescriptions

Figure 17 shows the percentage of acute laryngitis/tracheitis patients treated with narrow spectrum antibiotics (i.e., ATC = J01CE) among those who were treated with antibiotics. A higher value indicates that a majority of the cases were treated with the recommended antibiotics. An increase in the prescriptions of narrow-spectrum antibiotics also indicates a decrease in the prescriptions of broad-spectrum antibiotics and vice versa.

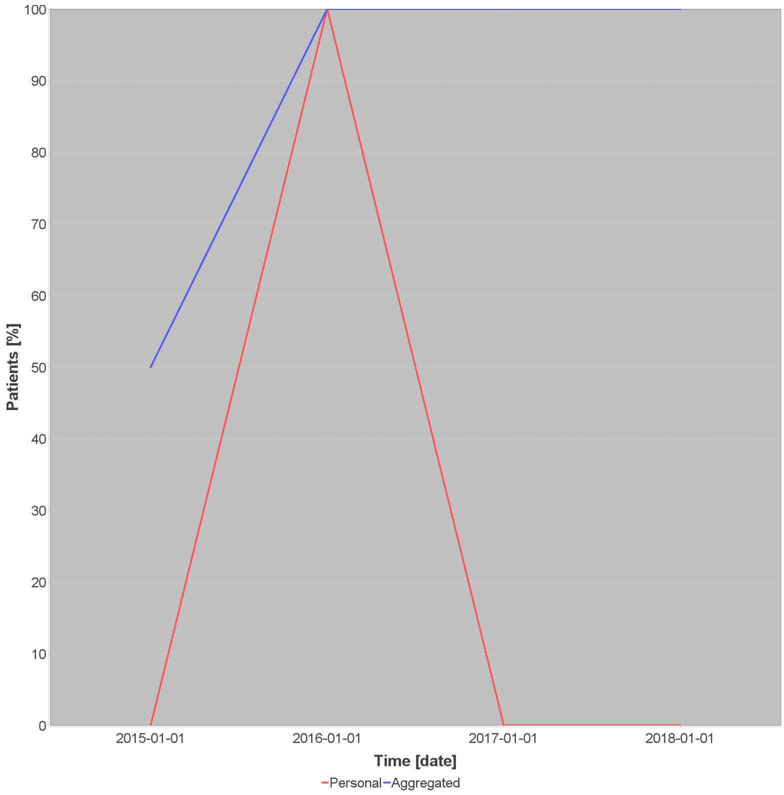

Figure 17: The percentage of narrow spectrum antibiotics (i.e., ATC = J01CE) of all antibiotics prescribed for acute laryngitis/tracheitis patients.

2.7. Unspecified respiratory infection

This section presents antibiotics consumption statistics for unspecified respiratory infection (i.e., ICPC2 = R83). Table 7 shows the number of your patients diagnosed with unspecified respiratory infection as well as the number of those treated with antibiotics, narrow spectrum antibiotics (i.e., ATC = J01CE) and broad spectrum antibiotics (i.e., ATC = J01A, J01C except J01CE, J01D, J01E, J01F or J01M).

Table 7: The number of your patients diagnosed with unspecified respiratory infection.

Time period		Patients diagnosed	Patients treated with antibiotics		
From	To		Total	Narrow spectrum	Broad spectrum
2015-01-01	2015-12-31	31	0	0	0
2016-01-01	2016-12-31	8	1	0	1
2017-01-01	2017-12-31	1	0	0	0
2018-01-01	2018-12-31	37	5	2	3

2.7.1. Total antibiotics prescriptions

Figure 19 shows the percentage of your patients treated with antibiotics among those who were diagnosed with unspecified respiratory infection.

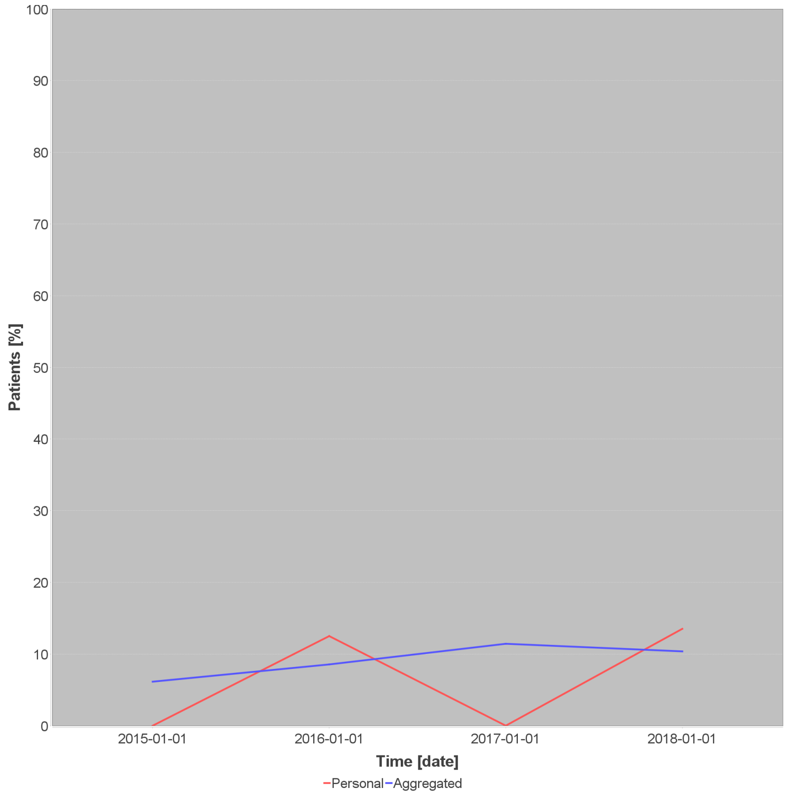

Figure 19: The percentage of patients diagnosed with unspecified respiratory infection treated with antibiotics.

2.7.2. Narrow spectrum antibiotics prescriptions

Figure 20 shows the percentage of unspecified respiratory infection patients treated with narrow spectrum antibiotics (i.e., ATC = J01CE) among those who were treated with antibiotics. A higher value indicates that a majority of the cases were treated with the recommended antibiotics. An increase in the prescriptions of narrow-spectrum antibiotics also indicates a decrease in the prescriptions of broad-spectrum antibiotics and vice versa.

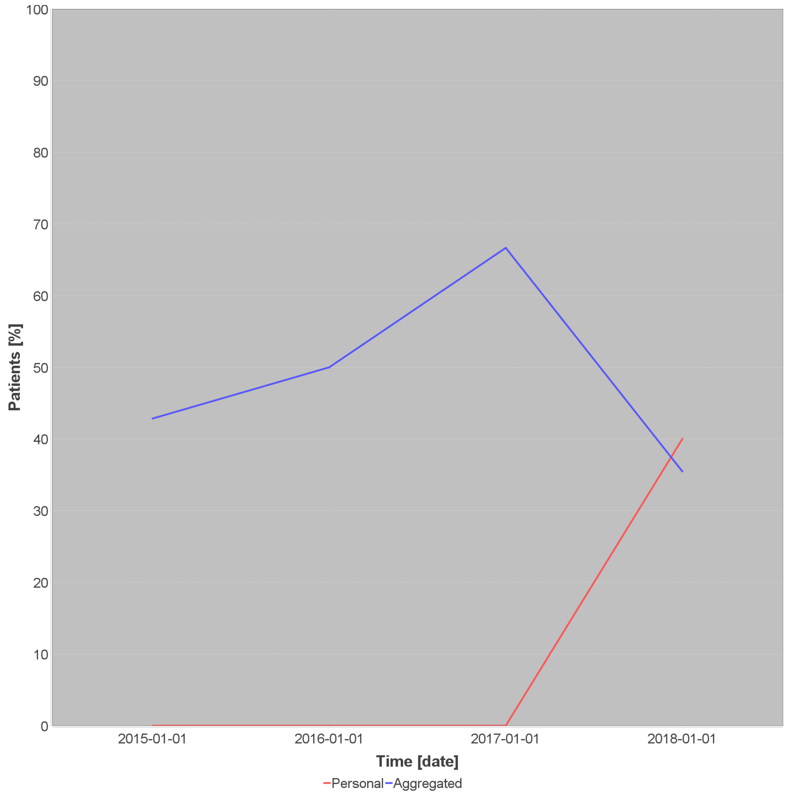

Figure 20: The percentage of narrow spectrum antibiotics (i.e., ATC = J01CE) of all antibiotics prescribed for unspecified respiratory infection patients.

3. Appendix

3.1. Indicators

The indicators used for measuring antibiotics prescription were developed by European Surveillance of Antimicrobial Consumption (ESAC).
N. Adriaenssens, S. Coenen, Disease-specific antibiotics prescribing quality indicators report, University of Antwerp, Antwerp, 2010.
https://bit.ly/2tGKMp3

3.2. ATC codes

This section provides a brief description of the ATC codes (J01D, J01C, J01A, J01E, J01F and J01M) used in the report. The complete ATC codes can be found at https://www.whocc.no/atc_ddd_index/.

- J01D - This group comprises beta-lactam antibacterials, other than penicillins.
- J01C - This group comprises penicillin beta-lactam antibacterials, including J01CE (Beta-lactamase sensitive penicillins), inhibiting bacterial cell wall synthesis.
- J01A - Tetracyclines
- J01E - This group comprises systemic sulfonamide and trimethoprim preparations.
- J01M - This group comprises quinolone antibacterials, inhibiting the bacterial DNA-gyrase.
- J01F - This group comprises macrolide, lincosamide and streptogramin antibacterials inhibiting bacterial protein synthesis through binding to the 50-S part of the ribosomes.
